# Aortic Graft Infection: Graphene Shows the Way to an Infection-Resistant Vascular Graft

**DOI:** 10.3389/fsurg.2017.00025

**Published:** 2017-05-04

**Authors:** Nikolaos Patelis, Dimitrios Schizas, Theodoros Liakakos, Chris Klonaris

**Affiliations:** ^1^First Department of Surgery, Vascular Unit, Medical School, National and Kapodistrian University of Athens, Laiko General Hospital, Athens, Greece

**Keywords:** graphene, aortic aneurysm, graft survival, infection, bacterial infections

## Abstract

Aortic graft infection is a potentially lethal complication of open and endovascular repair of aortic aneurysms. Graphene is the only existing two-dimensional material, and its unique structure gives graphene and its derivatives a plethora of original characteristics. Among other characteristics, graphene demonstrates bacteriostatic and bactericidal effects that could potentially resolve the problem of graft infection in the future. Data already exist in literature supporting this antibacterial effect of graphene oxide and reduced graphene oxide. Combining these materials with other substances enhances the antibacterial effect. Additionally, it looks feasible to expect antibiotic-delivering graphene-based graft materials in the future. Based on already published data, we could conclude that regarding graphene and its derivatives, the blessing of bactericidal effect comes with the curse of human cells toxicity. Therefore, it is important to find a fine balance between the desired antibacterial and the adverse cytotoxic effect before graphene is used in graft materials for humans.

Prosthetic graft infection is a grave and frequently lethal complication of both open and endovascular repair of aortic aneurysms (AA). Despite its relatively low overall incidence, the absolute numbers of graft infection are rising, as the use of synthetic grafts (Dacron, PTFE, or nylon) has become an everyday occurrence in both elective and emergency AA repair, with a reported graft infection incidence of 0.2 to as high as 6% in EVAR cases (1–4). Overall morbidity and mortality rates are high, and in cases of patients’ readmission due to aortic graft infection, in-hospital mortality can be 18% or higher (5, 6). Additionally, bacterial resistance to antibiotics is an evolving problem that could bring medicine and humanity back into the pre-antibiotic era if it remains unsolved (7).

Treatment of aortic graft infection varies from conservative treatment with long-term antibiotic administration to graft excision combined with a bypass procedure. The latter could be either extra-anatomical or follows the natural anatomic course of the aorta, but both approaches are linked to significant morbidity, mortality, and procedure-related complications. The *in situ* reconstruction of the aorta using either cryopreserved allografts or autologous veins is a similarly disabling method of aortic reconstruction after infected graft excision (8). As a result, our surgical attempts to resolve a case of aortic graft infection often bring our patient to worse status than before. The father of medicine, Hippocrates, stated that prevention is better than cure; therefore, the need for infection-resistant graft materials is essential in order to avoid the devastation of aortic graft infection and the patient disabling nature of existing treatment strategies.

Creating an infection-resistant material has been in the scope of biomedical engineering for a period of time, but focus has been concentrated on how an infection can be fought after it had occurred. Antibiotic-soaked and silver-containing grafts have already been used for AA repair after graft infection. Despite these grafts being a useful tool in the vascular surgery armament, the final outcomes of their use in real practice are ambiguous (9, 10). Other approaches, such as the use of bio-absorbable antibiotic-impregnated beads, have also been suggested as a potential solution to the problem of aortic graft infections (11).

Newly developed materials may cover the need for new infection-resistant graft materials. One of these futuristic super-materials that have already found its place in many health-related applications is graphene. Graphene is the only two-dimensional material, with each graphene layer being 1 atom or 0.345-nm thick (12). Graphene demonstrates a number of unique characteristics, and its promising future applications have awarded its creators, Andre Geim and Konstantin Novoselov, the 2010 Nobel Prize in Physics. Graphene is approximately more than 300-fold stronger than structural steel or Kevlar, 1,000-fold lighter than paper and almost as flexible as PTFE. These impressing characteristics are the result of the graphene’s structure as a carbon allotrope with sp2-bonded carbon atoms arranged in a sheet-like structure.

Graphene sheets are produced in a number of different methods, the most common of which are mechanical or thermal exfoliation, chemical vapor deposition (CVD), and epitaxial growth (12). Further post-processing of graphene sheets produce its derivatives, such as graphene oxide (GO) and reduced graphene oxide (rGO). Each of these methods and final products has slightly different characteristics and properties. Only graphene products of the highest quality demonstrate the abovementioned properties. These pristine graphene sheets are products of CVD, which at present renders the production expensive, but the price of graphene derivatives is constantly decreasing as production methods are optimized (13–15).

In existing literature, pristine graphene sheets have already been reported to have bacteriostatic and bactericidal properties. These two properties are of great interest for bioengineers committed to improve the graft materials of today to become less prone to bacterial infection. In a recent detailed review of graphene’s future in the grafts materials engineering, it seems that despite existing data supporting these beneficial properties of graphene, not all publications agree to the extent and efficacy of these (12). In this review, it has been reported that the surface of the graphene sheet plays a major role on the bactericidal action of this material. If the material is produced by vacuum filtration and the material particles lied flat on the surface, the bactericidal effect was minimum or none (16, 17). On the other hand, rGO with sharper particles’ edges demonstrated a significant bactericidal effect on *S. aureus* and *E. coli* bacteria (18–20). The mechanism behind this interaction of surface and bacteria is probably the bacterial membrane damage by the graphene edges and the efflux of cytoplasm. This proposed mechanism is also supported by the increased bactericidal effect of graphene on bacteria with thinner peptidoglycan membrane layer (21). At present, further mechanisms of actions on molecular level are also being considered, especially when combined materials are used (22–24).

In addition to the nature of the material surface, binding other molecules to graphene can also affect its bactericidal or bacteriostatic effect. Silver particles, platinum particles, chitosan, polyvinyl-*N*-carbazole, lactoferrin, diazonium salt, poly-l-lysine, and polyhexamethylene guanidine hydrochloride are a few of the substances used to increase the bactericidal and bacteriostatic effects of graphene sheets (12, 25–27). The results of these combined materials depend not only on the graphene derivative used (mainly GO or rGO) but also on the studied bacteria that show different interaction with these modified graphene surface. Endovascular materials are usually primarily contaminated and infected with *S. aureus* and *S. epidermidis*, although secondary hematogenic contamination with Gram-negative bacteria is also possible. rGO shows the strongest antibacterial effect toward both Gram-positive and Gram-negative bacteria, but in order to achieve the best possible antibacterial effect against the usual bacteria involved, further studies have to examine various combinations of graphene and other substances, although some evidence already exist (18).

Graphene has been used as a delivery agent for medication since 2008 (28). Since then graphene and its derivatives have been used in delivering a wide range of medication (29–33). In one case, graphene sheets were modified to deliver medication at a concentration controlled through electric pulses (34). Taking all these under consideration, it is not fiction to believe that in the future a graphene-based stent-graft could release antibiotics into the blood stream and/or the surrounding vessel wall when specific bacteria react with the graphene surface. This futuristic model would fight bacteria whenever that is necessary without contributing to bacteria multidrug resistance and by delivering the antibiotic directly to the affected tissues, thus minimizing systemic side effects.

Infection-resistant graphene-based graft materials could be constructed today with the already existing knowledge and technology. In the near future, these same graft materials could go a step further and evolve into infection fighting antibiotic-delivery agents. Unfortunately, before we see these steps ahead of current bioengineering, the question of biocompatibility and biotoxicity should be resolved (35). Graphene and its derivatives have been found to be toxic to certain lines of eukaryotic cells, and toxicity levels are analog to the bactericidal effect (24, 36, 37). From already published data, we could conclude that regarding graphene and its derivatives the blessing of bactericidal effect comes with the curse of human cells toxicity. Wrapping graphene particles within other structures or biofilms could resolve the problem of biotoxicity to some extent, but it would also affect its bactericidal effect (12, 38).

Graphene and its derivatives could potentially lead the way to new and truly infection-resistant or bactericidal graft materials that can be primarily used in aortic or other arterial repair in order to avoid the occurrence of graft infection, not fighting an already-occurring infection. A proper animal model and *in vivo* studies by a multidisciplinary team of bioengineers and vascular surgeons are necessary in order to research the potential role of graphene and its derivatives in building infection-resistant aortic grafts and provide the necessary data on patency, biotoxicity, and biocompatibility. These future materials would further reduce the incidence of graft infection that remains a lethal complication of open and endovascular aortic repair.

## Author Contributions

NP—conception, data collection, literature review, manuscript writing, and critical appraisal. DS—data collection and critical appraisal. TL—conception and critical appraisal. CK—conception, manuscript writing, and critical appraisal. All the authors have accepted the final version of the manuscript.

## Conflict of Interest Statement

The authors declare that the research was conducted in the absence of any commercial or financial relationships that could be construed as a potential conflict of interest.
